# Structural and functional properties of genes involved in human cancer

**DOI:** 10.1186/1471-2164-7-3

**Published:** 2006-01-11

**Authors:** Simon J Furney, Desmond G Higgins, Christos A Ouzounis, Núria López-Bigas

**Affiliations:** 1 Computational Genomics Group, The European Bioinformatics Institute, EMBL Cambridge Outstation, Cambridge CB10 1SD, UK; 2 Conway Institute of Biomolecular and Biomedical Research, University College Dublin, Belfield, Dublin 4, Ireland; 3 Genome Bioinformatics Laboratory. Center for Genomic Regulation, Universitat Pompeu Fabra, Pg. Maritim de la Barceloneta 37-49, E-08003, Barcelona, Spain

## Abstract

**Background:**

One of the main goals of cancer genetics is to identify the causative elements at the molecular level leading to cancer.

**Results:**

We have conducted an analysis of a set of genes known to be involved in cancer in order to unveil their unique features that can assist towards the identification of new candidate cancer genes.

**Conclusion:**

We have detected key patterns in this group of genes in terms of the molecular function or the biological process in which they are involved as well as sequence properties. Based on these features we have developed an accurate Bayesian classification model with which human genes have been scored for their likelihood of involvement in cancer.

## Background

All cancers are caused by alterations in DNA that affect the biochemical function or expression of certain genes providing expansion capabilities to the cell with the mutations. Generally this is a multi-step process, requiring mutations in several genes that ultimately result in the uncontrolled growth of a clone derived from the cells with the mutations[1]. A main aim in cancer research is to identify the causative genes and mutations leading to carcinogenesis. This knowledge can then be translated into new targets for diagnosis and treatment. The continuing investigation into the genetic basis of cancer has revealed a number of genes whose individual or concerted actions, when mutated, result in oncogenesis. Cancer-causing genes have been classified into three distinct groups: proto-oncogenes, tumour-suppressor genes, and stability genes, according to the biological roles they fulfil in a normal cell and hence, the aberrant process they effect in an oncogenic state[2]. Proto-oncogenes, when mutated, unleash their oncogenic potential primarily by remaining in a permanently activated state. On the other hand, oncogenic induction by tumour-suppressor genes occurs through the inactivation of the gene/protein. Stability genes are responsible for processes including DNA repair and chromosomal segregation. Mutations in these genes lead to a higher mutation rate in the genome[3].

The computational era of cancer research has revolved around the identification of transcriptomic differences between normal and cancerous tissues[4], and between tumour subtypes [5-7]. This field has been dominated by the analysis of microarray data to elucidate these differences[8]. Other studies have endeavoured to identify and examine orthologues of human cancer genes [9-11]. Recently, a census of human cancer genes was compiled[12]. This list, comprising 291 genes, is exclusively restricted to genes which, when mutated, are responsible to the development of cancer. In addition, the study recorded the mutation type evident in the cancer gene (somatic, germline, or both), neoplasm types associated with the gene (leukaemias/lymphomas, mesenchymal, epithelial, others), the phenotypic nature of the mutated gene (dominant or recessive), and the mechanism of mutation affecting each gene (e.g. translocation, deletion, frameshift). It has been suggested that 5–10% or more genes in the human genome could be contributing to oncogenesis[7]. Hence it is expected that many more genes involved in the cancer process remain to be identified[12].

Cancer is a complex disease with many different clinical forms and a relatively large number of genes involved. However, it has been suggested that, notwithstanding its complexity, cancer could be understood in terms of a small number of underlying principles[1]. Probably most, or perhaps all, types of human cancers show alterations in a small number of molecular, biochemical and cellular traits[1]. We have examined structural, functional and evolutionary properties of the group of causative genes of cancer as a whole, in order to unveil any common features and to uncover differences between this group of proteins and the entire human proteome.

Our analysis examines the distribution of Gene Ontology (GO) annotations[13] in the group of cancer genes compared to the rest of human proteins[14] to delineate trends in the biology of the oncoproteins. We have also analysed sequence properties of the cancer genes, such as the extent of conservation, paralogy and the protein and gene length, based on the hypothesis that these parameters influence the susceptibility of the genes to suffer alterations that could lead to a cancer phenotype. Since most of the genes in the cancer dataset analysed were identified by positional cloning without any previous hypothesis of biological function[12], we expect minimal biases due to the analysis of candidate genes with similar function or domains to the previously identified genes. Only a minority of known cancer genes were identified through analysis of plausible candidates based on known biological features of cancer cells[12].

If we assume that the trends observed in the group of known cancer genes reflect the general trends in all genes involved in oncogenesis, we should consider other genes in the human genome with similar trends as candidate genes involved in cancer development. We devised a model to identify and score such candidate cancer-related genes.

## Results

### Sequence properties of genes mutated in cancer cell

#### Degree of conservation

An examination of the level of conservation of cancer proteins compared to the rest of human proteins was facilitated by calculating the conservation score (cs) of these proteins in eukaryotic completed genomes (*Pan troglodytes*, *Mus musculus*, *Rattus norvegicus*, *Gallus gallus*, *Fugu rubripes*, *Danio rerio*, *Drosophila melanogaster*, *Anopheles gambiae*, *Caenorhabditis elegans *and *Caenorhabditis briggsae*) as described elsewhere[15] (see methods for details). Conservation scores (cs) range from 0, when no homologue is detected, to 1, when the closest homologue is identical to the human protein. This score is indicative of how conserved a protein has remained through evolution, and hence the degree to which mutations within the sequence are tolerated. Proteins involved in cancer show on average higher conservation scores than that of the human proteome in each of the species comparisons (Table 1).

**Figure 1:**
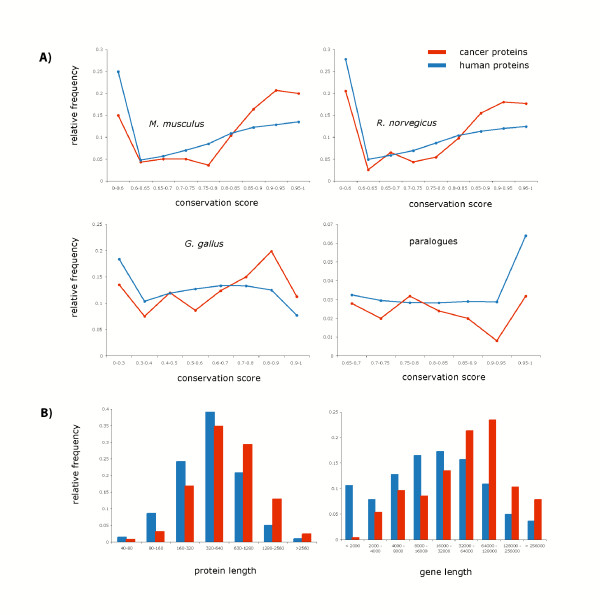
(a) Distribution of conservation score of proteins involved in cancer (red line) and all human proteins (blue line) against their closest homologue in *M. musculus*, *R. norvegicus*, *G. gallus *and between Paralogues. The conservation score gives an estimation of the mutation rate that the protein has been subjected to during evolution that is independent of the length of the protein. (b) Protein length, calculated as number of amino acids, and gene length distribution of cancer proteins (red) and all human proteins (blue).

In addition, the distributions of conservation scores between the cancer protein and human proteome datasets are markedly different (Figure 1; Table 1 for statistical analysis). It is evident in Figure 1 that a greater frequency of cancer proteins have high conservation scores (>0.8) compared to the human proteome. In fact, 67% of cancer proteins have conservation scores greater than 0.8 in mouse, whereas only 46% of the human proteome have scores in this range. Similar patterns are evident in the *Rattus *(61% of cancer proteins cs >0.8; 42% human proteome) and *Gallus *(31% of cancer proteins cs >0.8; 17% human proteome) proteomes.

Furthermore, when examining the degree of conservation within the cancer protein dataset, a fundamental division between proteins with dominantly and recessively acting mutations (according to the Cancer Census Database[12]) identifies a distinct pattern in the comparison proteomes. Proteins whose mechanism of cancer induction is caused by a dominant phenotype are more conserved than proteins that require a recessive phenotype to effect an oncogenic state (e.g. *M. musculus *average cs is 0.80 for dominant and 0.76 for recessive and *G. gallus *average cs is 0.64 for dominant and 0.56 for recessive, Supplementary Table 1).

#### Paralogy

To estimate the degree of paralogy within the human proteome, conservation scores for each human protein against its closest paralogue were calculated. These scores indicate whether or not a protein has a similar human homologue. Sufficiently close paralogues may possess a functionality similar enough to a cancer-causing protein to rescue a system from a disease state[16]. Cancer proteins have an average conservation score (0.36) lower than that of the human proteome (0.40; Table 1). In addition, a lower proportion of cancer genes have a conservation score >0.7 (12%) when compared to the human proteome (21%).

However, this view is reflective of the oncoprotein dataset as a whole and obscures an underlying trend in the paralogy properties of dominantly and recessively acting cancer proteins (Supplementary Table 1). When divided accordingly, dominant cancer proteins (n = 219) have an average conservation score of 0.41, in comparison to a conservation score of 0.19 for recessive proteins (n = 63). Furthermore, 14% of dominant cancer proteins possess a paralogue with a conservation score >0.7, compared to 5% of recessive proteins.

#### Length

Cancer genes are longer, on average, than genes from the remainder of the human genome (Fig. 1 and Table 2). Also the proteins encoded by the genes involved in cancer are, in general, longer than the rest of the human proteins (Fig. 1 and Table 2). Furthermore, when we split the cancer genes into those that are translocated in human cancers and those that register point mutations (according to the Cancer Census Database[12]), we observe an interesting pattern. The group of genes in which point mutations have been detected show on average longer coding sequences than translocated genes. In contrast, the translocated genes possess longer gene sequences than cancer genes with point mutations (Table 2).

### Function and process of cancer genes

**Figure 2:**
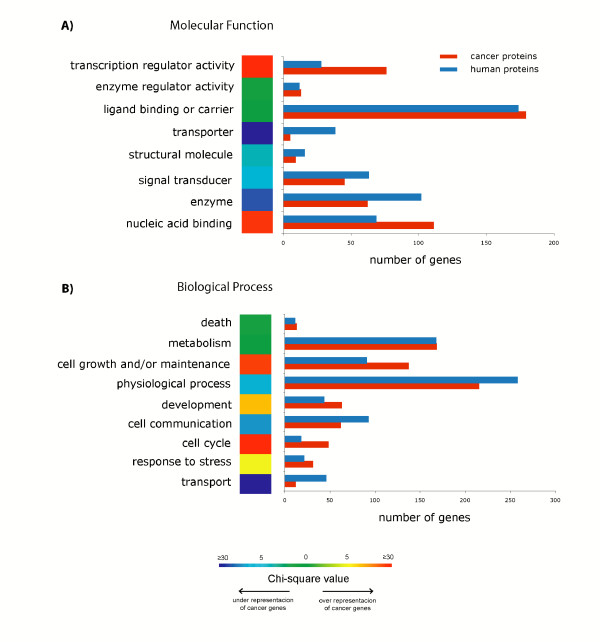
Number of genes involved in cancer with each Molecular function (a) or Biological process (b) GO assignments (red) and number of genes expected in a same size random group of genes from the human genome (blue) (the P-value for the χ^2 ^test is 1.5e-30 for the Molecular function and 3.5e-36 for the Biological process GO assignments). Note that one gene can have multiple GO assignments. χ^2 ^values for each cell are represented with a colour-coded scale. Colours towards red signify over-representation and those towards blue signify under-representation of cancer genes with a particular GO assignment. Green signifies equal representation of both sets in a category.

Gene Ontology (GO) terms have been used previously to characterise protein function and to elucidate trends in protein datasets[17]. We classified all human genes according to the molecular function of each protein and the biological process in which it is involved, as dictated by the Gene Ontology "slim" terms[13]. In total, 12222 human genes had a GO term assignment, of which 240 belonged to the cancer gene dataset. Analysis of the relative representations of both molecular functions and biological processes reveals particular trends in the cancer gene group compared to the human genome (Figure 2).

Transcription regulator activity and nucleic acid binding are significantly over-represented in the cancer genes, with transporter and enzyme function noticeably under-represented (Figure 2A). In terms of GO biological process, cancer genes, as expected, appear to be over-represented in cell cycle, cell-growth and/or maintenance, and developmental processes, whilst being considerably under-represented in transport processes (Figure 2B). Interestingly, 22 out of 30 of the cancer genes involved in stress response, and 27 out of 49 cancer genes involved in cell cycle show recessively acting mutations. For the other biological processes, higher proportions of genes belonging to the dominantly acting group are evident.

Table 3 lists the GO terms that are most significantly over- and under-represented in the cancer proteins. GO:0045786 (Negative regulation of cell cycle) is the most prominent disproportionately represented term. Interestingly, of the 22 cancer genes with this GO term (Table 3), 20 belong to the group that are prone to recessive mutations. This term describes only 46 further genes in the human genome. GO terms associated with the regulation of transcription, and kinase activity are most frequently over-represented amongst cancer proteins. GO terms depicting catalytic activity, transport and membrane integrality are notably under-represented.

### Bayesian method for the identification of genes likely to be involved in cancer

Based on the differences detected between genes involved in cancer and the rest of genes in the human genome, we wished to identify which other genes in the human genome are more likely to be involved in the cancer process. We developed and tested a naive Bayesian classifier based on sequence properties of the genes and the molecular function and biological processes in which they are involved.

Naive Bayes is a simple probabilistic induction algorithm widely used for classification problems[18,19]. This classifier learns from training data the conditional probability of each attribute given the class label. Classification is then done by applying Bayes rule[19] to compute the probability of the class for a particular instance in which the attributes are known[18].

We have applied the naive Bayes model to identify human genes likely to be involved in the cancer process based on sequence properties and the molecular function and biological process in which the genes are involved (based on GO terms). In particular, the attributes used to build the model are the assignment or non-assignment to 106 GO terms, the length of the protein and the length of the gene, the conservation score of the protein in eukaryotic completed genomes (*Pan troglodytes*, *Mus musculus*, *Rattus norvegicus*, *Gallus gallus*, *Fugu rubripes*, *Danio rerio*, *Drosophila melanogaster*, *Anopheles gambiae*, *Caenorhabditis elegans *and *Caenorhabditis briggsae*) and conservation score in paralogues. The length values and the conservation scores are used in the model as continuous features, while the GO terms are discrete features (1 or 0). The 106 GO terms used in the model were selected by computing the χ^2 ^value of each GO term with respect to the number of cancer genes assigned to the term compared to all human genes. Only those GO terms with a χ^2 ^value greater than 3 were used.

Although the positive set of genes from the cancer census can be generally trusted, producing negative sets for genes that are known not to be involved in cancer is not possible. Thus, to generate the negative examples, we randomly selected genes from the human genome that presumably are not known to be involved in cancer. However, a small proportion of these genes may well be involved in oncogenesis, although this property has not been detected yet. By implication, some of the false positive predictions might represent true positives – indeed, this is the predictive power of our current inductive approach.

To build the model, 100 sets of 480 genes were used: each with the 240 genes known to be involved in cancer and with GO terms assigned and a different set of 240 genes randomly selected from the group of 11982 human genes with GO terms assigned and not known to be involved in cancer. The final model used is the result of averaging the probabilities given by each of the 100 different models.

**Figure 3:**
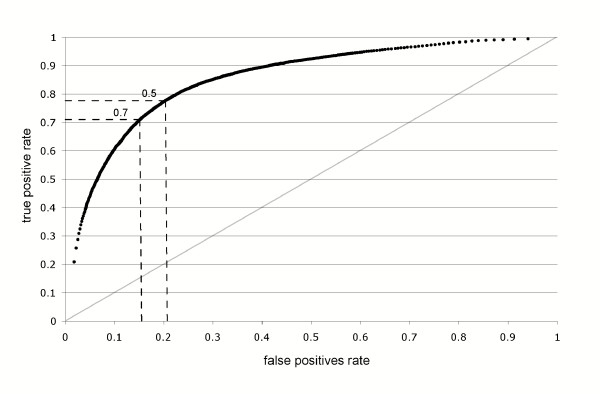
ROC curve for the prediction of cancer genes. The 45° diagonal of the ROC space represents a random guess situation. The performance of the model at 0.5 and 0.7 cut-off probability scores are shown with dashed lines.

Each of the models was validated with a 10-fold cross-validation test. This test consist of building the model with a fraction of the data (90%, learning set) and checking how well the model is able to predict the remaining fraction that has not seen before (10%, test set). This test was performed 10 times for each of the 100 sets of 480 proteins: on average, we obtained 78.1% accuracy, 79.2% specificity and 76.5% sensitivity. These values were calculated with a cut-off probability score of 0.5. The accuracy of the method was evaluated using an ROC (receiver operating characteristic) analysis (Figure 3) (see Methods for details).

We have applied this model to all the genes in the human genome with GO terms assigned (12222) and in total 2295 human genes are predicted with a probability score greater than 0.5 to be involved in cancer and 199 with a probability > 0.99 (Supplementary Table 2). We also list the 30 genes predicted with the highest probability score (Supplementary Table 3). All the genes predicted as cancer genes and the corresponding probability scores assigned by our method can be accessed via WWW [20].

## Discussion

### Sequence properties of cancer genes

The work presented here reveals that the group of genes involved in oncogenesis differs from the rest of human genes in sequence properties (conservation, paralogy and gene and protein length). It appears that the evolution of proteins causally involved in cancer is more tightly controlled than the human proteome in general (Figure 1). This is consistent with biological expectation: mutations, which can be disease-causing, are not readily tolerated in cancer proteins. A similar conservation pattern has been observed in a group of genes involved in hereditary disease[15]. Furthermore, proteins whose mechanism of cancer induction is dominant are more conserved than proteins that require a recessive phenotype to effect an oncogenic state. It is conceivable that a greater selective pressure is imposed on proteins in which mutation of a single allele leads to a dominantly phenotypic disease state. Conversely, it would follow that there is less selective pressure on a protein that requires mutations in both alleles to induce a cancer phenotype.

A low proportion of cancer proteins have highly conserved paralogs (Figure 1), this would indicate that the roles of proteins that become defective in cancer are less likely to be compensated for by wild-type paralagous proteins, as has been previously described for hereditary disease genes[15]. However this pattern is much more prominent in recessive cancer proteins. This is compatible with the fact that recessive mutations are generally loss-of-function mutations and functionality could be restored by the presence of a close paralogue. This is clearly not evident in a cancer disease state. Dominant mutations are predominantly gain-of-function or dominant-negative mutations for which a close paralogue would be unable to revert the biological perturbation.

Finally, cancer genes and proteins are longer, on average, than the rest of human genes. A similar pattern has been noticed in a comparison of proteins involved in hereditary disease[15]. Furthermore, the group of genes in which point mutations have been detected show on average longer coding sequences than translocated genes. In contrast, the translocated genes possess longer gene sequences than cancer genes with point mutations. This can be attributed to differences in the mutation process of these two groups of genes. In cancer, as in hereditary disease, a longer coding sequence is more susceptible to the acquisition of point mutations solely as a consequence of its length, and hence is more likely to produce a dysfunctional gene product. On the other hand, a longer gene sequence has a greater probability of being involved in a random translocation, and thus is more likely to produce a chimaeric gene implicated in oncogenesis.

In conclusion the sequence properties shown by the cancer genes are very similar to those previously described for genes involved in hereditary disease[15]. This is biologically relevant, as it is understood that the molecular mechanism that yields both groups of genes to cause either cancer or a hereditary disease is a mutation or alteration that impairs the normal functionality of the protein or modifies its expression. The sequence properties exhibited by this group of genes simply make them more likely to suffer these types of mutations.

### Function and process of cancer genes

The differential distribution of certain GO annotations in the group of cancer genes delineates trends in the functions and biological processes of the genes whose altered function or expression results in oncogenesis. Transcription regulator activity and nucleic acid binding are significantly over-represented in the cancer genes, with transporter and enzyme function noticeably under-represented (Figure 2A). This observation is attributable to the number of transcription factors that have been causally implicated in cancer (e.g. p53, c-myc, n-myc, pax3, pax8). In terms of GO biological process, cancer genes are over-represented in cell cycle, cell-growth and/or maintenance, and developmental processes, whilst are considerably under-represented in transport processes (Figure 2B). This result is consistent with the idea suggested by Hanahan and Weinberg that although the complexity of the cancer process, most human cancers would show alterations in a small number of molecular or cellular processes[1].

Although in this work we have focused on the analysis of the functions and processes in which cancer genes are involved, it would be also interesting to explore other type of data when available, for instance, the gene expression pattern of these genes or their genomic distribution. Also important is the fact that proteins interact between them or with DNA, and perform their function in the context of the cell and not individually, it would be therefore, interesting to investigate the involvement of cancer proteins in the context of protein networks and gene regulatory networks to get further knowledge of the tumorigenic process and improve on the prediction of cancer genes.

### Identifying genes likely to be involved in cancer

The unique pattern in GO annotation and sequence properties of cancer genes gives us the opportunity to identify which other genes in the human genome follow this pattern and thus are more likely to be altered in cancerous cells. We have developed a model using a Bayesian approach that is able to identify candidate genes for cancer.

We want to point out that both sequence properties and GO annotations are important for the correct identification of candidate genes for cancer. When we only use the GO annotations to build the Bayesian model, the sequence properties of the genes identified with a high likelihood of being involved in cancer differ from the sequence properties of cancer genes (i.e. the protein length, conservation and paralogy are similar to the rest of genes of the human genome and not to the cancer genes, see Supplementary Table 4 for details). This shows that it is not only the function of a gene nor the process in which it is involved that are indicative of its potential oncogenicity but that it is also a consequence of a gene's susceptibility to mutation which governs its liability to cause cancer. This also shows that the different sequence properties observed in the group of known cancer genes are not due to the fact that they belong to particular classes of genes, but due to their increased probability of suffering dysfunctional mutations solely as a consequence of their sequence properties (i.e. protein length, conservation and paralogy).

The 30 genes predicted with the highest probability score by our method are listed in Table 3. Of these, some have been found to be implicated in cancer although they are not included in the Cancer Census Dataset (see supplementary Table 5). Four of the genes (Nuclear factor NF-kappa-B p100/p49 subunits, MYST histone acetyltransferase 3, C-ets-1 protein (p54) and C-ets-2 protein) have been implicated in cancer-causing translocations [21-25]. In addition, Hypermethylated in cancer 1 protein (Hic-1) has been reported to be underexpressed in tumour cells due to hypermethylation and in mice, heterozygous disruption of the gene has been shown to induce tumours[26,27]. The complete list of genes predicted as cancer genes and the corresponding probability scores assigned by our method can be accessed via WWW [20]. We believe that this information could facilitate the process of finding the causative mutations or alterations in different cancer types.

## Conclusion

In summary, we have analysed the sequence and functional properties of the group of genes known to be causative of cancer when mutated. We have detected clear trends in this group of genes in terms of the molecular function or the biological process in which they are involved as well as sequence properties. Based on these features we have developed an accurate Bayesian classification model with which human genes have been scored for their likelihood of involvement in cancer. The results can be consulted by WWW [20].

## Methods

### Data

The list of genes involved in cancer was obtained from the Cancer Gene Census Database [28]. This list comprises 291 genes, and is exclusively restricted to genes which, when mutated, are responsible to the development of cancer.

All human genes were classified according to the molecular function of each protein and the biological process in which they are involved according to the Gene Ontology "slim" terms[13].

### Computation of conservation score

Conservation score (cs) is a measure that gives an estimation of the mutation rate that the protein has been subjected to during evolution that is independent of the length of the protein[15]. This was computed using WUBLASTP (version 2.0)[29], which is based on the public domain NCBI BLAST version 1.4[30]. Hits with E_values > 10^-10 ^were discarded. Smith-Waterman[31] alignment was performed on the pairs that gave a significant BLAST hit. The value of cs was calculated for each human gene as the WUBLASTP score of the closest homologue in each eukaryotic completed genome (*Pan troglodytes*, *Mus musculus*, *Rattus norvegicus*, *Gallus gallus*, *Fugu rubripes*, *Danio rerio*, *Drosophila melanogaster*, *Anopheles gambiae*, *Caenorhabditis elegans *and *Caenorhabditis briggsae*) divided by the WUBLASTP score of the protein against itself.

### Naive Bayes model

We have applied the naive Bayes model to identify human genes likely to be involved in the cancer process based on sequence properties and the molecular function and biological process in which the genes are involved (based on GO terms). This classifier learns from training data the conditional probability of each attribute given the class label. Classification is then done by applying Bayes rule[19] to compute the probability of the class for a particular instance in which the attributes are known[18].

The attributes used to build the model are the assignment or non-assignment to 106 selected GO terms (terms with a χ^2 ^value greater than 3), the length of the protein and the length of the gene, the conservation score of the protein in eukaryotic completed genomes (*Pan troglodytes*, *Mus musculus*, *Rattus norvegicus*, *Gallus gallus*, *Fugu rubripes*, *Danio rerio*, *Drosophila melanogaster*, *Anopheles gambiae*, *Caenorhabditis elegans and Caenorhabditis briggsae*) and conservation score in paralogues.

The model was build by averaging the probabilities given by 100 different models, each built with 240 genes known to be involved in cancer and with GO terms assigned and a different set of 240 genes randomly selected from the group of 11982 human genes with GO terms assigned and not known to be involved in cancer.

Each of the models was validated with a 10-fold cross-validation test. This test consist of building the model with a fraction of the data (90%, learning set) and checking how well the model is able to predict the remaining fraction that has not seen before (10%, test set). This test was performed 10 times for each of the 100 sets of 480 proteins.

We use an ROC curve to evaluate the overall accuracy and predictive value of the method. The ROC analysis is a standard approach to evaluate the sensitivity and specificity of prediction methods (Figure 3). It estimates a curve, which describes the inherent tradeoff between sensitivity and specificity of a model. Each point on the ROC curve is associated with a specific prediction criteria – in this case it is the cut-off probability score above which genes are considered candidates to be involved in cancer. The ROC curve is obtained by plotting the True Positive rate (fraction of known cancer genes that are predicted by the method) against the False Positive rate, for different values of the cut-off probability score. The 45° diagonal of the ROC space represents a random guess situation.

## Authors' contributions

SJF carried out the statistical studies, participated in the development of the prediction method and drafted the manuscript. DGH helped to draft the manuscript and revised it critically. CAO participated in the design of the study and helped to draft the manuscript. NLB conceived the study, participated in its design and coordination and helped to draft the manuscript. All authors read and approved the final manuscript.

## Supplementary Material

Additional File 1 it contains supplementary table 1, supplementary table 2, supplementary table 3, supplementary table 4 and supplementary table 5. Click here for file

## Figures and Tables

**Table 1:** Mean values and statistical analysis for degree of conservation and paralogy. Kolmogorov-Smirnov (KS) test of the conservation score between cancer proteins and the rest of human proteins. The KS test analyses show how different two distributions are, and computes a probability (P-value) that the two distributions are equal as well as the maximum distance (D) between them.

**Genome**	**Average cs**		**KS test: conservation score (cs)**	
	**Cancer genes**	**Non-cancer genes**	**D (%)**	**P-value**

M. musculus	0.79	0.73	19.5	1.57e-09
*R. norvegicus*	0.77	0.71	16.7	4.69e-07
*G. gallus*	0.62	0.56	14.1	5.36e-05
*F. rubripes*	0.52	0.48	10.6	5.4e-03
paralogues	0.36	0.40	9.8	9.1e-03

**Table 2:** Mean values and statistical analysis for gene length, protein length and the gene protein length ratio. The P-value for the KS test of the values distribution between each of the groups and the non-cancer group is shown in parenthesis.

	**Protein length**	**Gene length**	**Gene/protein length**
Cancer genes	721 (<2.2e-16)	87426 (5e-14)	157 (4.1e-08)
Cancer genes with point mutations	817 (1.2e-8)	82615 (7.4e-07)	121 (3.9e-03)
Translocated cancer genes	690 (7.7e-08)	92494 (8.7e-15)	176 (7.7e-08)
Non-cancer genes	491	49437	114

**Table 3:** Selected GO annotations of genes involved in cancer compared to all human genes. The sign in the χ^2 ^value indicates over-representation (positive values) or under-representation (negative values) of the GO term in the group of cancer proteins.

**GO id**	**GO term**	**#total**	**#cancer genes**	**χ^2^**
GO:0045786	negative regulation of cell cycle	68	22	225.91
GO:0003684	damaged DNA binding	35	10	88.55
GO:0030528	transcription regulator activity	1034	76	85.86
GO:0006355	regulation of transcription, DNA-dependent	1281	84	73.49
GO:0003700	transcription factor activity	770	59	72.73
GO:0007049	cell cycle	601	48	64.36
GO:0005634	nucleus	2492	122	47.13
GO:0006366	transcription from Pol II promoter	181	19	41.93
GO:0008151	cell growth and/or maintenance	3014	137	40.58
GO:0003713	transcription coactivator activity	109	13	35.30
GO:0006281	DNA repair	94	11	28.98
GO:0003676	nucleic acid binding	2546	111	27.88
GO:0003824	catalytic activity	3768	62	-14.46
GO:0006810	transport	1529	12	-20.14
GO:0016021	integral to membrane	1986	20	-20.31
