# Supplementary material for: Structural and functional properties of genes involved in human cancer
Source: BMC Genomics. 2006 Jan 11;7:3. doi: 10.1186/1471-2164-7-3 (PMC1373651; doi:10.1186/1471-2164-7-3)
Supplement: Additional File 1 — it contains supplementary table 1, supplementary table 2, supplementary table 3, supplementary table 4 and supplementary table 5. [file 1471-2164-7-3-S1.pdf]

Supplementary Material for:

Structural and functional properties of genes involved in human cancer

Simon J. Furney[1,#], Desmond G. Higgins[2], Christos A. Ouzounis[1,*] & Núria López-Bigas[1,*]

*For correspondence: N. L.-B or C.A.O.

fax +44-1223-492588 • email: nlbigas@ebi.ac.uk or ouzounis@ebi.ac.uk

Supplementary Table 1. Mean values for degree of conservation and paralogy for dominantly- and recessively- acting mutated cancer genes.

Genome	Average cs	
	Dominant	Recessive
M. musculus	0.80	0.76
R. norvegicus	0.78	0.74
G. gallus	0.64	0.56
F. rubripes	0.53	0.51
Paralogues	0.41	0.19

Supplementary Table 2. Number of genes predicted by the method to be involved in cancer with different probabilty scores.

Probability score	Total of predicted genes	Total of known cancer genes	Number of new candidate genes
> 0.5	2494	199	2295
>0.6	2134	189	1945
>0.7	1773	178	1595
>0.8	1403	165	1238
>0.9	967	147	820
>0.95	706	127	579
>0.99	283	84	199
>0.999	76	37	39

Supplementary Table 3. The top thirty genes predicted to be cancer-causing with p > 0.99 by the Bayesian classifier.

Ensembl gene ID	Description	Position
ENSG00000134954	C-ets-1 protein (ETS1)	11q24
ENSG00000070444	MAX binding protein (MNT)	17p13
ENSG00000073282	tumor protein p73-like (TP73L)	3q28
ENSG00000126733	dachshund 2 (DACH2)	Xq21
ENSG00000101057	Myb-related protein B (MYBL2)	20q13
ENSG00000077150	Nuclear factor NF-kappa-B p100/p49 subunits (NFKB2)	10q24
ENSG00000057657	PR-domain zinc finger protein 1 (PRDM1)	6q21
ENSG00000123933	Max-interacting transcriptional repressor (MXD4)	4p16
ENSG00000148516	Transcription factor 8 (TCF8)	10p11
ENSG00000077092	Retinoic acid receptor beta (RARB)	3p24
ENSG00000102974	Transcriptional repressor (CTCF)	16q22
ENSG00000122641	Inhibin beta A chain precursor (INHBA)	7p14
ENSG00000032514	DNA excision repair protein (ERCC-6)	10q11
ENSG00000116016	Hypoxia-inducible factor 2 alpha (EPAS1)	2p21
ENSG00000134250	Neurogenic locus notch homolog protein 2 precursor (NOTCH2)	1p11
ENSG00000157557	C-ets-2 protein (ETS2)	21q22
ENSG00000108264	Transcriptional adapter 2-like (TADA2L)	17q12
ENSG00000100146	Transcription factor (SOX-10)	22q13
ENSG00000179348	Endothelial transcription factor (GATA-2)	3q21
ENSG00000136574	Transcription factor (GATA-4)	8p23
ENSG00000177374	Hypermethylated in cancer 1 protein (HIC1)	17p13
ENSG00000115415	Signal transducer and activator of transcription 1 (STAT1)	2q32
ENSG00000186951	Peroxisome proliferator activated receptor alpha (PPARA)	22q13
ENSG00000107223	Endothelial differentiation-related factor 1 (EDF1)	9q34
ENSG00000121054	Nucleoside diphosphate kinase B (NME2)	17q21
ENSG00000137273	Forkhead box protein F2 (FOXF2)	6p25
ENSG00000116833	Hepatocytic transcription factor (NR5A2)	1q32
ENSG00000083168	MYST histone acetyltransferase 3 (MYST3)	8p11
ENSG00000151422	Proto-oncogene tyrosine-protein kinase (FER)	5q21
ENSG00000015171	Adenovirus 5 E1A-binding protein (ZMYND11)	10p15

Supplementary Table 4. Comparison of sequence properties of genes involved in cancer, genes predicted with probability score > 0.95 using sequence properties plus GO annotations (SEQ+GO), genes predicted with probability score > 0.95 using only GO annotations (GO) and all human genes.

	**Cancer genes**	**SEQ+GO**	**GO**	**All genes**
Gene length	87426	72858	48531	54754
Protein length	721	687	558	556.4
Paralogues	0.37	0.37	0.40	0.41
Mus musculus	0.80	0.84	0.80	0.75
Rattus norvegicus	0.77	0.81	0.76	0.72
Gallus gallus	0.58	0.60	0.53	0.53
Fugu rubripes	0.50	0.51	0.48	0.46
Number of genes	240	579	542	12192

Supplementary Table 5. Evidences for involvement in cancer for 15 of the 30 top scoring candidate genes for cancer.

Ensembl gene ID	Evidences for involment in cancer
ENSG00000015171	BS69 protein has been implicated as a tumour suppressor through its inhibition of adenovirus type 5 E1A 32 kDa protein and resultant stifling of adenovirus replication(1)
ENSG00000057657	Beta-interferon gene positive-regulatory domain I binding factor is a transcriptional repressor, a dysfunctional form of which has been found to be highly expressed in myeloma cell lines(2).
ENSG00000073282	p63 is critical for epithelial stem cell renewal and epithelial homeostasis, and along with p73 is required for p53-dependent apoptosis in response to DNA damage(3).
ENSG00000077092	The transcription factor retinoic acid receptor beta is a potent inhibitor of breast cancer cells in vitro and loss of expression is commonly observed during breast carcinogenesis(4).
ENSG00000077150	A B cell lymphoma-associated chromosomal translocation, t(10;14)(q24;q32), juxtaposes the immunoglobulin C alpha 1 locus to Nuclear factor NF-kappa-B p100/p49 subunits (Oncogene Lyt-10)(5).
ENSG00000083168	MYST histone acetyltransferase 3 is involved in acute myeloid leukemias through a chromosomal translocation(6).
ENSG00000102974	CTCF, which binds to a number of important regulatory regions within the 5' noncoding sequence of the human MYC oncogene, is localised at chromosome segment 16q22.1 within one of the smallest regions of overlap for common deletions in breast and prostate cancers(7)
ENSG00000115415	STAT 1. In one study spontaneous malignant tumors were found not to occur in wildtype mice, occur late in half of mice lacking either Rag2 or Stat1, but occur early in 82% of mice lacking both genes(8)
ENSG00000121054	Nucleoside diphosphate kinase B is implicated in control of the metastatic potential of malignant cells(9).
ENSG00000134954	C-ets-1 protein (p54) has been implicated in translocations in different forms of leukaemia(10,11), and to suppress tumorigenicity in human colon cancer cell lines(12).
ENSG00000136574	It has been suggested that GATA-4 has a relationship to tumorigenesis or tumor progression in somatic cell-derived testicular neoplasms(13).
ENSG00000157557	C-ets-2 protein has been implicated in translocations in different forms of leukaemia(11,14).
ENSG00000177374	Hypermethylated in cancer 1 protein (Hic-1) is reported to be ubiquitously expressed in normal tissues, but underexpressed in different tumour cells where it is hypermethylated(15). Recently it has been shown that mice disrupted in the germ line for only one allele of Hic1 develop many different spontaneous malignant tumors(16).
ENSG00000179348	A recent study has implicated endothelial transcription factor GATA-2 in myeloid leukaemia(17).
ENSG00000186951	PPARα mediates the hepatocarcinogenic effect of certain peroxisome proliferators in rodents. However, no carcinogenic effect of peroxisome proliferators has been found in humans, possibly because expression of PPARα is much lower in human liver than in rodent liver or due to other species-specific differences(18).

References:

1. Hateboer, G., Gennissen, A., Ramos, Y.F., Kerkhoven, R.M., Sonntag-Buck, V., Stunnenberg, H.G. and Bernards, R. (1995) *Embo J*, **14,** 3159-3169.
2. Gyory, I., Fejer, G., Ghosh, N., Seto, E. and Wright, K.L. (2003) *J Immunol*, **170,** 3125-3133.
3. Flores, E.R., Tsai, K.Y., Crowley, D., Sengupta, S., Yang, A., McKeon, F. and Jacks, T. (2002) *Nature*, **416,** 560-564.
4. Sirchia, S.M., Ren, M., Pili, R., Sironi, E., Somenzi, G., Ghidoni, R., Toma, S., Nicolo, G. and Sacchi, N. (2002) *Cancer Res*, **62,** 2455-2461.
5. Neri, A., Chang, C.C., Lombardi, L., Salina, M., Corradini, P., Maiolo, A.T., Chaganti, R.S. and Dalla-Favera, R. (1991) *Cell*, **67,** 1075-1087.
6. Borrow, J., Stanton, V.P., Jr., Andresen, J.M., Becher, R., Behm, F.G., Chaganti, R.S., Civin, C.I., Disteche, C., Dube, I., Frischauf, A.M. *et al.* (1996) *Nat Genet*, **14,** 33-41.
7. Filippova, G.N., Lindblom, A., Meincke, L.J., Klenova, E.M., Neiman, P.E., Collins, S.J., Doggett, N.A. and Lobanenkov, V.V. (1998) *Genes Chromosomes Cancer*, **22,** 26-36.
8. Shankaran, V., Ikeda, H., Bruce, A.T., White, J.M., Swanson, P.E., Old, L.J. and Schreiber, R.D. (2001) *Nature*, **410,** 1107-1111.
9. Backer, J.M., Mendola, C.E., Kovesdi, I., Fairhurst, J.L., O'Hara, B., Eddy, R.L., Jr., Shows, T.B., Mathew, S., Murty, V.V. and Chaganti, R.S. (1993) *Oncogene*, **8,** 497-502.
10. Diaz, M.O., Le Beau, M.M., Pitha, P. and Rowley, J.D. (1986) *Science*, **231,** 265-267.
11. Sacchi, N., Watson, D.K., Guerts van Kessel, A.H., Hagemeijer, A., Kersey, J., Drabkin, H.D., Patterson, D. and Papas, T.S. (1986) *Science*, **231,** 379-382.
12. Suzuki, H., Romano-Spica, V., Papas, T.S. and Bhat, N.K. (1995) *Proc Natl Acad Sci U S A*, **92,** 4442-4446.
13. Ketola, I., Pentikainen, V., Vaskivuo, T., Ilvesmaki, V., Herva, R., Dunkel, L., Tapanainen, J.S., Toppari, J. and Heikinheimo, M. (2000) *J Clin Endocrinol Metab*, **85,** 3925-3931.
14. Le Beau, M.M., Rowley, J.D., Sacchi, N., Watson, D.K., Papas, T.S. and Diaz, M.O. (1986) *Cancer Genet Cytogenet*, **23,** 269-274.
15. Wales, M.M., Biel, M.A., el Deiry, W., Nelkin, B.D., Issa, J.P., Cavenee, W.K., Kuerbitz, S.J. and Baylin, S.B. (1995) *Nat Med*, **1,** 570-577.
16. Chen, W.Y., Zeng, X., Carter, M.G., Morrell, C.N., Chiu Yen, R.W., Esteller, M., Watkins, D.N., Herman, J.G., Mankowski, J.L. and Baylin, S.B. (2003) *Nat Genet*, **33,** 197-202.
17. Wieser, R., Volz, A., Vinatzer, U., Gardiner, K., Jager, U., Mitterbauer, M., Ziegler, A. and Fonatsch, C. (2000) *Biochem Biophys Res Commun*, **273,** 239-245.
18. Kersten, S., Desvergne, B. and Wahli, W. (2000) *Nature*, **405,** 421-424.
